# Cyclin-Dependent Kinase Inhibitors as Marketed Anticancer Drugs: Where Are We Now? A Short Survey

**DOI:** 10.3390/molecules190914366

**Published:** 2014-09-11

**Authors:** Gaëlle Mariaule, Philippe Belmont

**Affiliations:** 1 Institut Curie, UMR CNRS 176, 26 rue d’Ulm, Paris 75005, France; E-Mail: gaelle.mariaule@curie.fr; 2 Faculté de Pharmacie de Paris, Université Paris Descartes, UMR CNRS 8638, 4 avenue de l’Observatoire, Paris 75006, France

**Keywords:** CDK, kinase, inhibitor, anticancer, heterocycle, clinical evaluation, ATP-competitive, allosteric site, ATP non-competitive

## Abstract

In the early 2000s, the anticancer drug imatinib (Glivec^®^) appeared on the market, exhibiting a new mode of action by selective kinase inhibition. Consequently, kinases became a validated therapeutic target, paving the way for further developments. Although these kinases have been thoroughly studied, none of the compounds commercialized since then target cyclin-dependent kinases (CDKs). Following a recent and detailed review on the subject by Galons *et al*., we concentrate our attention on an updated list of compounds under clinical evaluation (phase I/II/III) and discuss their mode of action as ATP-competitive inhibitors. CDK inhibition profiles and clinical development stages are reported for the 14 compounds under clinical evaluation. Also, tentative progress for forthcoming potential ATP non-competitive inhibitors and allosteric inhibitors are briefly described, along with their limitations.

## 1. Introduction

**Figure 1:**
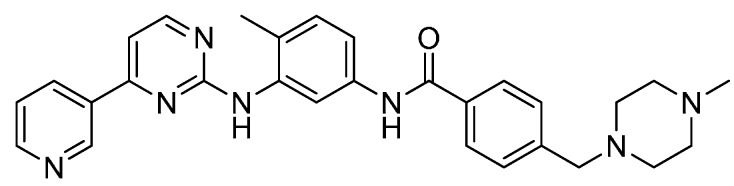
Imatinib (Glivec^®^), the first kinase inhibitor.

At the beginning of the 21st century, a breakthrough arose with the development of a tyrosine kinase inhibitor, imatinib (generic name stem*-ib* standing for inhibitor, Figure 1), as a new type of cancer drug. As a result, protein kinases became clearly validated drug targets for cancer therapy [1,2].

Protein kinases, through phosphorylation reactions, are implicated in the regulation of various cellular activities since they belong to the cellular signaling molecules and therefore regulate all the processes that are essential for growth, development and homeostasis of eukaryotic cells [1,3].

Although the key role of phosphorous in cellular metabolism has been known for more than a century, it was only in the 1950 that Burnett and Kennedy discovered the phenomenon of protein phosphorylation [1]. Since the 1980s the role of protein kinases in oncogenesis and tumor growth was clearly demonstrated, and the profound understanding of the regulation mechanism led to the Nobel Prize award to Krebs and Fischer in 1992 [1].

The human protein kinases set (kinome), is constituted of 518 identified proteins, divided in seven families [4]. Cyclin-Dependent Kinases (CDKs) are part of the CMGC family named after the members: *Cyclin-dependent kinases* (CDKs), *Mitogen-activated protein kinases* (MAPKs), *Glycogen synthase kinases* (GSKs) and *CDK-like kinases* (CLKs). The CDK sub-family comprises thirteen members (CDK1 to CDK13). For their discovery, Hartwell, Nurse and Hunt received the Nobel Prize in 2001 [5].

CDKs can be separated roughly in two groups, those that mediate cell progression (CDK1, CDK2, CDK3, CDK4, CDK6), and those that regulate transcription (CDK7-CDK9 and CDK11-CDK13). CDKs’ regulation is due to the association with a protein, a cyclin (25 members are known). It is noteworthy that the expression of CDKs remains quite constant, while cyclin levels vary throughout cell cycle. Abnormal expression of cyclins has a direct impact on cell deregulation that may lead to tumor development. Moreover, CDK11 and CDK12 are involved in the regulation of RNA splicing, and CDK5 and CDK10 in neuronal functions [6,7,8].

Overall, the regulation mechanism for CDKs is mediated by the association with cyclins which leads to several conformational modifications, for instance the 90° shift of CDKs PSTAIRE α-helix (see also Section 4) [9]. The PSTAIRE helix is an α-helix in the amino-terminal lobe of CDKs (also known as the α1 helix), which is moved inward upon cyclin binding, resulting in reorientation of key active-site residues (the name of this helix comes from its amino-acid sequence, which is conserved among all major CDKs) [9,10]. Some other regulation mechanisms are the activation/inhibition through phosphorylation [10,11,12] and also the action of inhibitory proteins (Cyclin-dependent Kinase Inhibitors or CKIs) due to their association with CDKs (e.g., p15, p16, p18, p19) or CDK/cyclin complexes (e.g., p21, p27, p57) [13,14].

This review will focus at first on ATP-competitive CDK inhibitors studied in the clinic, which still remain the most successful approach. In order to keep on with recent results we have cited when appropriate data reported for clinical trials on the National Institute of Health website. Then, we report briefly on ATP non-competitive CDK inhibitors (small molecules or small peptides) and finally we shed light on allosteric CDK inhibitors which is a burgeoning field of investigation.

## 2. ATP-Competitive Inhibitors

The great majority of known CDK inhibitors are ATP-competitive, interacting with cyclin-dependent kinases within their catalytic ATP-site. Until now, this strategy has been the most successful one in order to develop powerful inhibition of CDKs implicated in the cell cycle [1,15,16].

Many compounds have been patented due to their high inhibition profiles against CDKs, and they have been thoroughly reviewed [17,18,19,20,21], particularly those nowadays under clinical development [15,22,23]. The structures of these molecules are quite diverse and they are generally constituted or derived from various heterocyclic families such as purines, pyrimidines, indoles, pyrazoles, thiazoles, or derived from natural products such as staurosporine or flavones [16].

**Figure 2:**
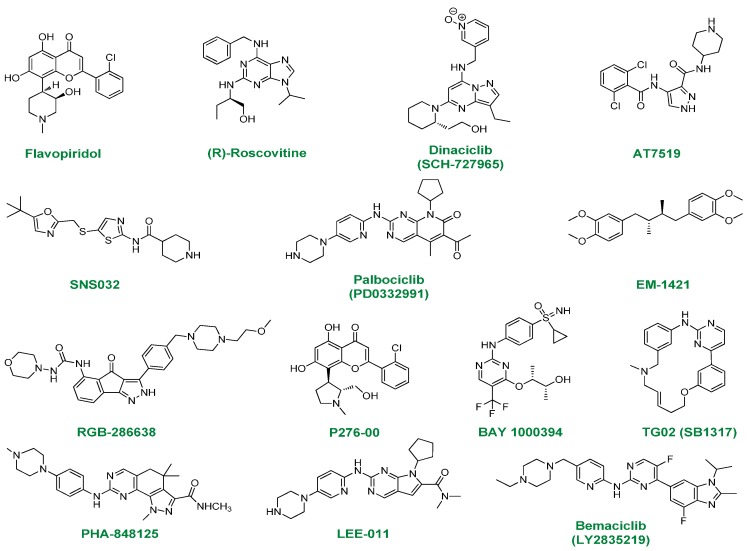
Cyclin-dependent kinase inhibitors (14 compounds) under clinical evaluation.

We have chosen to present only the molecules which are currently under clinical evaluation. To our knowledge, they are 14 compounds (Figure 2) at present at this stage (in comparison to a list of 11 reported recently) [22]. For some compounds (R-547, ZK-304709, AZD-5438, AG-24322) the clinical trials have been discontinued [21,22] and some were not treated in this review (e.g., UCN-01, a 7-hydroxystaurosporine derivative), due to several drawbacks such as suboptimal human pharmacokinetics (low solubility, high doses to be administered) or the lack of selectivity towards CDKs [1,20,21,22].

Among the CDKs inhibitors presented in Figure 2, we find flavopiridol [24,25] and roscovitine [26,27,28] which were the first generation CDK inhibitors to enter clinical trials for use in anticancer therapy. Clearly, their extensive study paved the way for the development of the second generation compounds.

A brief description for each compound presented in Figure 2 will be given below. Further, readers will also find a table summarizing the data for these drug candidates (Table 1).

**Table 1:** CDKs inhibitor in clinics: An overview.

Drug Candidate	Company	Administration Mode	CDK Inhibition Profile: IC_50_	Clinical Trial Stage
**Flavopiridol**	Sanofi-Aventis	intravenous	CDK1: 30 nM, CDK2: 100 nM CDK4: 20 nM, CDK6: 60 nM CDK7: 10 nM, CDK9: 10 nM	II
**Roscovitine**	Cyclacel	oral	CDK1: 2.7 μM, CDK2: 0.1 μM CDK7: 0.5 μM, CDK9: 0.8 μM	II
**Dinaciclib**	Merck	intravenous	CDK1: 3 nM, CDK2: 1 nM CDK5: 1 nM, CDK9: 4 nM	III
**SNS032**	Sunesis	intravenous	CDK2: 38 nM, CDK7: 62 nM CDK9: 4 nM	I
**AT7519**	Astex/Novartis	intravenous	CDK1: 190 nM, CDK2: 44 nM CDK4: 67 nM, CDK5: 18 nM CDK9: <10 nM	I/II
**PD0332991**	Pfizer	oral	CDK4: 11 nM, CDK6: 16 nM	III
**EM-1421**	Erimos	intravenous	CDK1: N/A	I/II
**RGB-286638**	Agennix	intravenous	CDK1: 2 nM, CDK2: 3 nM CDK3: 5 nM, CDK4: 4 nM CDK9: 1 nM	I
**P276-00**	Nicholas Piramal	intravenous	CDK9: 20 nM, CDK1: 79 nM CDK2: 224 nM, CDK4: 63 nM	II
**BAY-1000394**	Bayer	oral	CDK1-4, 7, 9: 5-25 nM;	I
**TG02/SG1317**	S*BIO/Tragara	oral	CDK9: 3nM, CDK5: 4 nM, CDK2: 5 nM, CDK3: 8 nM, CDK1: 9nM	I
**PHA-848125 AC**	Nerviano	oral	CDK1: 2 nM, CDK2: 3 nM CDK4: 5 nM, CDK5: 4 nM	II
**LEE-011**	Novartis/Astex	oral	CDK4, 6: N/A	III
**LY2835219**	Eli Lilly	oral	CDK4, 6: N/A	I/(III)

### 2.1. Flavopiridol

Flavopiridol, also called alvocidib (generic name stem *-cidib* standing for cyclin-dependent kinase inhibitor), is a flavonoid derived from an Indian plant, rohitukine. This ATP-competitive derivative was jointly developed by Sanofi-Aventis and the US National Cancer Institute (NCI) [1]. Flavopiridol has the potential to inhibit several CDKs having a key role in the cell cycle at submicromolar concentrations: CDK1 (IC_50_: 30 nM), CDK2 (IC_50_: 100 nM), CDK4 (IC_50_: 20 nM), CDK6 (IC_50_: 60 nM) and CDK7 (IC_50_: 10 nM). It also inhibits CDK9 (IC_50_: 10 nM), which plays a role in the regulation of the transcription of mRNA via the phosphorylation of RNA polymerase II. This compound is capable of stopping the cell cycle at two levels: either during phase G1, or during the switch between G2 phase to M phase [29].

Flavopiridol underwent phase II clinical trial for the treatment of acute myeloid leukemia along with chronic lymphoid leukemia. It was shown that flavopiridol also possesses a synergistic action with other antitumor agents such as docetaxel, irinotecan or cisplatin. The co-administration with another cytotoxic agent proved to be a useful tactic in order to reduce the amount of flavopiridol, thus limiting its side-effects [20,30].

However, Sanofi-Aventis seems to have abandoned the development of flavopiridol around 2010, or at least put it on stand-by [31], although a positive phase II trial for patients with acute myeloid leukemia just came out in 2014 with Tolero Pharmaceuticals, and was reported at the American Society of Clinical Oncology annual meeting [32]. Indeed, several studies have already demonstrated that induction therapy with flavopiridol (50 mg/m^2^ days 1–3), followed by cytarabine (667 mg/m^2^/days 6–8) and mitoxantrone (40 mg/m^2^ day 9) yields complete remission rates of nearly 70% in patients with newly diagnosed, poor-risk acute myeloid leukemia. This randomized phase II trial was performed in order to study how alvocidib, cytarabine, and mitoxantrone work compared to cytarabine and daunorubicin in treating patients with newly diagnosed acute myeloid leukemia. The results showed that mitoxantrone induction results in significantly higher complete remission rates compared with the other treatments [32].

### 2.2. (R)-Roscovitine

(*R*)-roscovitine, also called seliciclib (generic name stem *-ciclib* standing for cyclin-dependent kinase inhibitor as for *-cidib* above) or CYC202, was entered into clinical trials in 2001 by Cyclacel Pharmaceuticals, Inc. This derivative inhibits several CDKs with various IC_50_ values: CDK1 (2.7 μM), CDK2 (0.1 μM), CDK7 (0.5 μM) and CDK9 (0.8 μM) [1,33], but the inhibition activity of this compound is poor towards CDK4 and CDK6 (IC_50_ > 100 μM). (*R*)-roscovitine inhibits both the phosphorylation of retinoblastoma protein (pRb protein) and RNA polymerase II [1,34].

(*R*)-roscovitine is in phase II clinical trials and has been tested on lung cancer (non-small cell lung cancer, NSCLC). In other phase II studies seliciclib was administered in combination with gemcitabine and cisplatin as first-line treatment and with docetaxel as second-line treatment in NSCLC [35]. This compound is administered *per os* and demonstrated good bioavailability in phase I studies. Seliciclib is also being evaluated in a phase II study as a single agent in patients with nasopharyngeal cancer and displayed noticeable tumor shrinkage [35].

### 2.3. Dinaciclib

Dinaciclib, also called SCH-727965, is under study at Merck. It is a particularly potent inhibitor of CDK1 (IC_50_: 3 nM), CDK2 and CDK5 (IC_50_: 1 nM), and also of CDK9 (IC_50_: 4 nM). This derivative is in fact far more efficient than flavopiridol, because even if there is a poor selectivity within the CDKs, it exhibits an excellent selectivity towards other kinases, thus showing a better toxicity profile.

Dinaciclib has entered phase III clinical development for the treatment of chronic lymphocytic leukemia in 2012 [36], therefore no data are available yet, but this study is being conducted to demonstrate the superiority of dinaciclib compared to ofatumumab in chronic lymphocytic leukemia participants who are refractory to either fludarabine treatment or chemoimmunotherapy [36].

### 2.4. AT7519

AT7519 was advanced to the clinic by Astex Pharmaceuticals. It inhibits several CDKs, thus affecting cell cycle regulation, and is also a potent inhibitor of RNA polymerase II–dependent transcription. The IC_50_ values against CDKs are: CDK1 (IC_50_: 190 nM), CDK2 (IC_50_: 44 nM), CDK4 (IC_50_: 67 nM), CDK5 (IC_50_: 18 nM) and CDK9 (IC_50_: < 10 nM).

Novartis has an option to develop and commercialize AT7519, which is in phase I of clinical development for treating refractory solid tumors and is given intravenously [37]. In this phase I pharmacokinetic and pharmacodynamic study of AT7519, among the twenty-eight patients treated, one developed hypotension, and one died, but they were able to determine the dose-limiting toxic effects [37]. Two separate phase II trials have also been underway since 2012 for mantle cell lymphoma and chronic lymphocytic leukemia [38], but no further data are available.

### 2.5. SNS032

SNS032, previously called BMS-387032, has been developed by Sunesis. This compound, which contains a thiazole unit, selectively inhibits CDK2 (IC_50_: 38 nM), CDK7 (IC_50_: 62 nM) and CDK9 (IC_50_: 4 nM) [39]. Preclinical studies demonstrated that SNS032 was able to inhibit cell cycle activity along with transcription [20].

SNS032 is in phase I clinical trials for the treatment of chronic lymphoid leukemia along with multiple myeloma, and the mode of administration is intravenous [39]. The purpose is to evaluate the dose-escalation of SNS-032 along with its safety, pharmacokinetics, pharmacodynamic activity and clinical efficacy. Biomarker analyses demonstrated mechanism-based pharmacodynamic activity with inhibition of CDK7 and CDK9, although limited clinical activity in heavily pretreated patients was observed [39].

### 2.6. PD0332991

PD0332991, also called palbociclib, is the property of Pfizer. PD0332991 shows a strong inhibition selectivity against CDK4 (IC_50_: 11 nM) and CDK6 (IC_50_: 16 nM) [20]. This compound inhibits the phosphorylation of pRb protein which results in the cell cycle arrest at the G1 phase.

Several phase II clinical studies are ongoing or terminated on solid tumors. Indeed, Pfizer has already reported encouraging phase II data suggesting that its breast cancer candidate can extend progression-free survival by 18 months compared to standard therapy, therefore encouraging a planned phase III clinical trial for the treatment of breast cancer [31]. The blockbuster dreams seem very close [40].

### 2.7. EM-1421

EM-1421, often called terameprocol, is the property of the pharmaceutical company Erimos. It inhibits CDK1 [41]. EM-1421 seems to be still in phase I/II clinical trials for the treatment of patients with refractory solid tumors [22]. A phase I study is also conducted in order to determine the safety, maximum tolerated dose, dose limiting toxicity, pharmacokinetics (clearance from the blood) of EM-1421 given to patients with leukemia, as intravenous infusion three times a week [42].

### 2.8. RGB-286638

RGB-286638, developed by Agennix, also inhibits several CDKs in the low nanomolar IC_50_ range: CDK1 (IC_50_: 2 nM), CDK2 (IC_50_: 3 nM), CDK3 (IC_50_: 5 nM), CDK4 (IC_50_: 4 nM) and CDK9 (IC_50_: 1 nM).

RGB-286638 has completed phase I clinical development for the treatment of multiple myeloma and solid tumors [43], but a planned phase I clinical trial for relapsed or refractory hematological malignancies was withdrawn in 2012 prior to enrollment [44]. Moreover, Agennix announced on May 2013 its dissolution and that the liquidation should take place in 2014, therefore we have no clear vision for the future development of RGB-286638 [45].

### 2.9. P276-00

P276-00 is an analog of flavopiridol where the piperidine moiety has been changed for a pyrrolidine and resulted from an intense structure-activity and co-crystallization effort [24,46,47]. P276-00 inhibits efficiently CDK9 (IC_50_: 20 nM), but also other CDKs such as CDK1 (IC_50_: 79 nM), CDK2 (IC_50_: 224 nM) and CDK4 (IC_50_: 63 nM) [24,47].

The development is being performed by Nicholas Piramal Company and phase II clinical trials for various types of tumours have been announced (e.g., recurrent and/or locally advanced head and neck cancer, relapsed and/or refractory mantle cell lymphoma) [48]. Although several phase II trials ended in 2012/2013 no results could be found.

### 2.10. BAY-1000394

BAY-1000394, developed by Bayer, inhibits CDKs involved in the cell cycle (CDK1, CDK2, CDK3 and CDK4) along with the one implicated in the regulation of transcription (CDK7 and CDK9), with IC_50_ values ranging from 5 to 25 nM.

This compound is in phase I clinical development for various types of tumours. BAY-1000394 studies also shown that it could be efficient in combination with cisplatin [49]. BAY 1000394 clearly overcomes many limitations of other drugs since it displays high solubility in water, even at neutral pH, and low efficacious oral doses. Furthermore, BAY 1000394 has proven antitumor activity in xenograft models resistant to standard drugs such as doxorubicin, cisplatin, or paclitaxel, and has shown its potential for combination treatment with drugs on the market [49]. The full story about this nanomolar pan-CDK inhibitor BAY 1000394, which contains an unusual sulfoximide group, has been recently disclosed [50].

### 2.11. TG02

TG02/SG1317, is a pyrimidine-based derivative that inhibits CDKs together with JAK2 and FLT3 [51]. TG02 induces G1 cell cycle arrest and apoptosis in a broad range of tumor cell lines. TG02, originally designated SB1317, was discovered by S*BIO and licensed to Tragara Pharmaceuticals in 2008. The *in vitro* kinase spectrum of TG02 is CDK9 (IC_50_: 3 nM), CDK5 (IC_50_: 4 nM), CDK2 (IC_50_: 5 nM), CDK3 (IC_50_: 8 nM) and CDK1 (IC_50_: 9 nM) [52].

TG02 was selected for phase I clinical trials [53] and the results are expected in 2014 for the phase 1 study in patients with chronic lymphocytic leukemia and small symphocytic symphoma [54] and in 2015 for phase I study in patients with advanced hematological malignancies [55].

### 2.12. PHA-848125

PHA-848125 AC, also called milciclib, developed by Nerviano, is selective towards several CDKs: CDK1 (IC_50_: 2 nM), CDK2 (IC_50_: 3 nM), CDK4 (IC_50_: 5 nM), CDK5 (IC_50_: 4 nM), and is in phase II clinical trials for treating thymic carcinoma [56]. The results of phase II for milciclib in patients with thymic carcinoma have been reported at the 2014 American Society of Clinical Oncology (ASCO) annual meeting [57]. Indeed, 43 patients have been treated and the second stage of the trial is still ongoing. Out of 30 patients whose data are available, 14 are successes with a progression free survival rate at 3 months of 46.7%, the toxicity being generally moderate [57]. Recently, a patent was filled for the treatment of mesothelioma [58].

### 2.13. LEE-011

**Figure 3:**
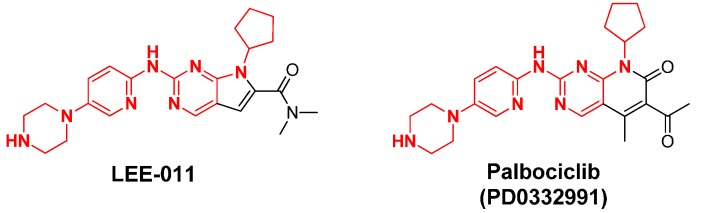
Comparison of Astex/Novartis’ LEE-011 and Pfizer’s PD0332991 structures.

LEE-011 is one of the most selective inhibitors for CDK4 and CDK6 [59] and is being developed by Astex Pharmaceuticals™ and Novartis. In January 2014 this inhibitor entered phase III clinical trials for the treatment of breast cancer [60]. Due to encouraging results LEE-011 has now become the main competing drug-candidate with Pfizer’s PD0332991 (palbociclib), see Figure 3 [59].

Upon comparison of the chemical structure of Novartis’ LEE-011 and Pfizer’s PD0332991, the similarity is evident. The major difference lies in the bicyclic core since LEE-011 possesses a pyrrolo-pyrimidine and PD0332991 a pyridopyrimidine. The “*east*” part of the structure is also modified. The structural similarities make their analogous CDKs inhibition profiles (high selectivity for CDK4 and CDK6) quite obvious Moreover, both derivatives are orally administered which is pretty advantageous compared with dinaciclib, which is also in phase III clinical trials but is administered intravenously.

### 2.14. LY2835219

LY2835219, also called abemaciclib, is also a selective CDK4/CDK6 inhibitor, blocking cells at G1 phase due to the inhibition of the phosphorylation of pRb protein. Abemaciclib has been developed by Eli Lilly and is in phase I clinical trials for treating non-small-cell lung carcinoma. LY2835219 already demonstrated very good results for patients suffering from breast cancer and a phase I trial in combination with hormone therapies for breast cancer will finish in 2016 [61]. A phase III study of LY2835219 combined with fulvestrant for women with HER2-negative metastatic breast cancer is planned in 2014 [62].

### 2.15. An Overview

Table 1 represents an overview of the data detailed above. Indeed, for every drug candidate, the company involved is listed along with the administration mode, the CDKs targeted and finally the actual clinical trials status. In general, all drug candidates have planar structures constituted by heterocycles or hydrophobic aromatic rings. Usually, they are poorly selective between the different CDKs and even against other kinases. This low selectivity induces difficulties for drug dosing during the clinical studies, and the use of higher doses results in toxicity. On the contrary, if lower doses are used they become inefficient. Therefore, one way to avoid side effects is to combine these drug candidates with classical cytotoxic agents. This process proved to be very efficient in the case of some inhibitors such as flavopiridol or BAY-1000394. This poor selectivity is also the sign that there is a high degree of homology between all the CDKs, especially within the ATP-binding site.

## 3. ATP Non-Competitive Inhibitors

Compounds belonging to this family are a new inhibitor generation which does not enter in competition with ATP, therefore exhibiting an action mode different from the other type of inhibitors. In fact, finding inhibitors following this strategy is a recent tactic, so very few molecules have been identified and to date none of them have entered clinical stage [30]. Two classes of ATP non-competitive CDKs inhibitors have been identified:

### 3.1. Small Molecules Inhibitors

**Figure 4:**
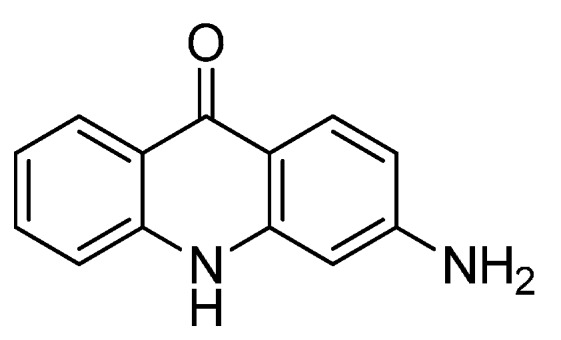
3-Aminothioacridinone (3-ATA).

These small molecules inhibitors have been essentially identified by their IC_50_ values on CDK/cyclin complexes in the presence of high ATP concentration. Therefore, these small molecules have the ability to inhibit the CDK/cyclin complex without interacting with the ATP-binding site. One typical example of such small molecule inhibitors is 3-aminothioacridinone (3-ATA), discovered by Kelley’s group [63], see Figure 4.

Kelley’s team demonstrated that 3-ATA was able to inhibit CDK4/cyclinD complex by interaction with a site different from the ATP-binding site. Furthermore, this represents an interesting therapeutic strategy for patients presenting a tumor p16 deficit. Indeed, when p16 protein is in deficit, the CDK cannot be inactivated, therefore inducing a deregulation in the cell cycle leading to tumor genesis [64]. Moreover, 3-ATA exhibited very interesting *in vitro* inhibition towards CDK4/cyclinD (3.1 μM). Additionally, it was shown that this inhibitor did not affect CDK4/p16 interaction, suggesting that it is not a p16-mimic. Remarkably, *in vitro* tests revealed that 3-ATA had a greater inhibitory activity on tumor cells compared to normal cells. Among the compounds screened with 3-ATA, two derivatives (BTD and NSC 625987) were shown to inhibit *in vitro* cyclin D1 binding to CDK4 at nearly 1000-fold higher concentration than their IC_50_ on CDK4 kinase, indicating that for these compounds direct inhibition of cyclin D1 binding to CDK4 was not the central mechanism for CDK4 kinase activity inhibition [63]. This study is of great interest since a lead compound, 3-ATA, was identified, with specific CDK4/cyclinD complex inhibitory properties. It is also noteworthy that the deregulation in cancer of cyclins D and their ability to activate CDK4 and CDK6, also makes them an attractive therapeutic target [65].

### 3.2. Small Peptide Inhibitors

The second class of ATP non-competitive CDKs inhibitors includes small peptides designed based on their similarity with endogenous CDKs inhibitors, such as p21, p27 and p57 [30].

Seminal work of Kaelin’group [66] led the way for the conception of small peptides inhibitors. Indeed, they studied the interaction of the CDK2/cyclin complex with several cell cycle regulators and they were able to identify a common recognition motif for these CDKs regulators (including the endogenous CDKs inhibitors: p21, p27 and p57) [67]. This eight-residue peptide motif displays a highly conserved amino-acid sequence and the peptides containing this sequence are able to inhibit the activity of CDK2/cyclin complex. Since this seminal work several peptides having this motif have been developed in order to mimic the endogenous CDKs inhibitors [30,68,69,70].

In addition, to create a more drug-like and less peptidic compound, development of the REplacement with Partial Ligand Alternatives through Computational Enrichment (REPLACE) strategy was used in order to generate inhibitors of CDKs/cyclin interactions [71]. Thanks to this method, fragment optimization could be successfully obtained, in particular for compounds targeting CDK2 substrate recruitment site on the cyclin regulatory subunit. Indeed, inhibition of CDKs through the cyclin provides an approach to obtain selectivity against other protein kinases and to specifically block the activity of the G1- and S-phase CDKs, since they are the only ones containing a functional cyclin binding groove. The most potent compound identified so far exhibits an IC_50_ of 18.1 μM against CDK2/cyclinA [71].

## 4. Allosteric Inhibitors

The inhibitors of this family have the specificity to link CDKs away from their ATP-binding site. The fixation on the allosteric sites will induce a conformational change to the CDKs, thus modifying their activity. These inhibitors have the advantage to be more selective since they exploit a specific environment for each CDK. This seems a promising strategy for CDKs since it has already been successful for other protein kinases such as Abl, p38 and MEK1 [72,73].

**Figure 5:**
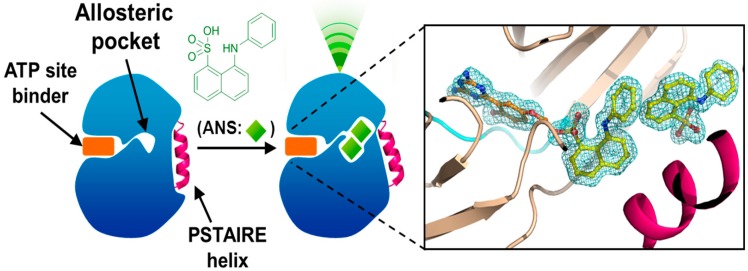
CDK2 allosteric site [74] (reproduction/modification by courtesy of E. Schönbrunn).

To our knowledge, no CDK inhibitors of this type have been created yet. However, it is noteworthy that the team of Schönbrunn has discovered a potential allosteric site for CDK2 protein [74]. For this purpose of identifying an allosteric site which could therefore alter the interaction between the CDK and the cyclin, they have explored CDK2 protein with a fluorophore 1-AnilinoNaphthalene-8-Sulfonic acid (1,8-ANS). Gratifyingly, thanks to spectroscopy fluorescence analysis, an area within the CDK2 itself has been identified (Figure 5).

Furthermore, using co-crystallization data, Schönbrunn’s group observed that two ANS molecules were able to interact with CDK2 in a different site from the ATP-binding site. More importantly, once the ANS molecules (in green, Figure 5) are linked to CDK2, a change of conformation of the PSTAIRE helix (in magenta, Figure 5) occurred, thus rendering impossible the association with the cyclin.

## 5. Conclusions

ATP non-competitive inhibitors and allosteric inhibitors strategies are of great interest since they should allow access to more selective inhibitors. However, the main drawback is still to know which CDK or CDK/cyclin complex should be selectively targeted. There are still many details to understand about the implication of CDKs in the cell cycle and their precise implication in tumor growth. The lack of selectivity of CDKs inhibitors tested up to now is a disadvantage since we still cannot draw clear conclusions for the inhibition of a specific CDK. Moreover, Schönbrunn’s group has shown recently that the acetyllysine binding site of bromodomain-containing protein 4 (BRD4) could interact with diverse kinase inhibitors (e.g., dinaciclib and flavopiridol) [75,76]. This observation suggests that this protein family could be an off-target of kinase inhibitors; therefore this dual inhibition could be profitable for the design of a new generation of inhibitors.

Nevertheless, designing ATP-competitive inhibitors is still the most studied approach. Drug candidates such as dinaciclib, PD0332991 and LEE-011 are leading the way since they are in phase III clinical trials. These three compounds are the most advanced in the study of CDK inhibition as a treatment for cancers, but there are still no drugs on the market for which the therapeutic target is proven to be CDKs. Therefore this field of research remains an intriguing and exciting one!

## References

[1] Matthews D.J., Gerritsen M.E. (2010). Targeting Protein Kinases for Cancer Therapy.

[2] Moen M.D., McKeage K., Plosker G.L., Siddiqui M.A. (2007). Imatinib: A review of its use in chronic myeloid leukaemia. Drugs.

[3] Meijer L. (2003). Le cycle de division cellulaire et sa régulation. Oncologie.

[4] Manning G., Whyte D.B., Martinez R., Hunter T., Sudarsanam S. (2002). The protein kinase complement of the human genome. Science.

[5] Schwob E. (2001). Nobel Prize in Medicine 2001: The universal key to cell division. Bull. Cancer.

[6] Malumbres M., Barbacid M. (2005). Mammalian cyclin-dependent kinases. Trends Biochem. Sci..

[7] Satyanarayana A., Kaldis P. (2009). Mammalian cell-cycle regulation: Several CDKs, numerous cyclins and diverse compensatory mechanisms. Oncogene.

[8] Kohoutek J., Blazek D. (2012). Cyclin K goes with CDK12 and CDK13. Cell Div..

[9] De Bondt H.L., Rosenblatt J., Jancarik J., Jones H.D., Morgan D.O., Kim S.H. (1993). Crystal structure of cyclin-dependent kinase 2. Nature.

[10] Morgan D.O. (1995). Principles of CDK regulation. Nature.

[11] Solomon M.J. (1993). Activation of the various cyclin/CDC2 protein kinases. Curr. Opin. Cell Biol..

[12] Karlsson-Rosenthal C., Millar J.B. (2006). CDC25: Mechanisms of checkpoint inhibition and recovery. Trends Cell Biol..

[13] Elledge S.J., Winston J., Harper J.W. (1996). A question of balance: The role of cyclin-kinase inhibitors in development and tumorigenesis. Trends Cell Biol..

[14] Kaldis P. (1999). The CDK-activating kinase (CAK): From yeast to mammals. Cell. Mol. Life Sci..

[15] Galons H., Oumata N., Meijer L. (2010). Cyclin-dependent kinase inhibitors: A survey of recent patent literature. Expert Opin. Ther. Pat..

[16] Sharma P.S., Sharma R., Tyagi R. (2008). Inhibitors of cyclin dependent kinases: Useful targets for cancer treatment. Curr. Cancer Drug Targets.

[17] Cicenas J., Valius M. (2011). The CDK inhibitors in cancer research and therapy. J. Cancer Res. Clin. Oncol..

[18] Fischer P.M., Gianella-Borradori A. (2003). CDK inhibitors in clinical development for the treatment of cancer. Expert Opin. Investig. Drugs.

[19] Canavese M., Santo L., Raje N. (2012). Cyclin dependent kinases in cancer: Potential for therapeutic intervention. Cancer Biol. Ther..

[20] Diaz-Padilla I., Siu L.L., Duran I. (2009). Cyclin-dependent kinase inhibitors as potential targeted anticancer agents. Investig. New Drugs.

[21] Lapenna S., Giordano A. (2009). Cell cycle kinases as therapeutic targets for cancer. Nat. Rev. Drug Discov..

[22] Galons H., Oumata N., Gloulou O., Meijer L. (2013). Cyclin-dependent kinase inhibitors closer to market launch?. Expert Opin. Ther. Pat..

[23] Stone A., Sutherland R.L., Musgrove E.A. (2012). Inhibitors of cell cycle kinases: Recent advances and future prospects as cancer therapeutics. Crit. Rev. Oncog..

[24] De Azevedo W.F., Mueller-Dieckmann H.J., Schulze-Gahmen U., Worland P.J., Sausville E., Kim S.H. (1996). Structural basis for specificity and potency of a flavonoid inhibitor of human CDK2, a cell cycle kinase. Proc. Natl. Acad. Sci. USA.

[25] Christian B.A., Grever M.R., Byrd J.C., Lin T.S. (2007). Flavopiridol in the treatment of chronic lymphocytic leukemia. Curr. Opin. Oncol..

[26] De Azevedo W.F., Leclerc S., Meijer L., Havlicek L., Strnad M., Kim S.H. (1997). Inhibition of cyclin-dependent kinases by purine analogues: Crystal structure of human CDK2 complexed with roscovitine. FEBS J..

[27] Meijer L., Raymond E. (2003). Roscovitine and other purines as kinase inhibitors. From starfish oocytes to clinical trials. Acc. Chem. Res..

[28] Whittaker S.R., Walton M.I., Garrett M.D., Workman P. (2004). The Cyclin-dependent kinase inhibitor CYC202 (*R*-roscovitine) inhibits retinoblastoma protein phosphorylation, causes loss of Cyclin D1, and activates the mitogen-activated protein kinase pathway. Cancer Res..

[29] Kaur G., Stetler-Stevenson M., Sebers S., Worland P., Sedlacek H., Myers C., Czech J., Naik R., Sausville E. (1992). Growth inhibition with reversible cell cycle arrest of carcinoma cells by flavone L86–8275. J. Natl. Cancer Inst..

[30] Abate A.A., Pentimalli F., Esposito L., Giordano A. (2013). ATP-noncompetitive CDK inhibitors for cancer therapy: An overview. Expert Opin. Investig. Drugs.

[31] Guha M. (2012). Cyclin-dependent kinase inhibitors move into Phase III. Nat. Rev. Drug Discov..

[32] Zeidner J.F., Foster M.C., Blackford A., Litzow M.R., Morris L., Strickland S.A., Lancet J.E., Bose P., Levy M.Y., Tibes R. Randomized multicenter phase II trial of timed-sequential therapy with flavopiridol (alvocidib), cytarabine, and mitoxantrone (FLAM) versus “7+3” for adults with newly diagnosed acute myeloid leukemia (AML). Proceedings of the American Society of Clinical Oncology Conference.

[33] McClue S.J., Blake D., Clarke R., Cowan A., Cummings L., Fischer P.M., MacKenzie M., Melville J., Stewart K., Wang S. (2002). *In vitro* and *in vivo* antitumor properties of the cyclin dependent kinase inhibitor CYC202 (*R*-roscovitine). Int. J. Cancer.

[34] Meijer L., Borgne A., Mulner O., Chong J.P., Blow J.J., Inagaki N., Inagaki M., Delcros J.G., Moulinoux J.P. (1997). Biochemical and cellular effects of roscovitine, a potent and selective inhibitor of the cyclin-dependent kinases CDC2, CDK2 and CDK5. FEBS J..

[35] Cyclacel.com. http://www.cyclacel.com/research_programs_oncology_cyc202.shtml.

[36] A Phase 3 Study Comparing Dinaciclib *versus* Ofatumumab in Patients with Refractory Chronic Lymphocytic Leukemia (P07714 AM2). http://clinicaltrials.gov/ct2/show/NCT01580228?term=dinaciclib&rank=14.

[37] i>Mahadevan D., Plummer R., Squires M.S., Rensvold D., Kurtin S., Pretzinger C., Dragovich T., Adams J., Lock V., Smith D.M. (2011). A phase I pharmacokinetic and pharmacodynamic study of AT7519, a cyclin-dependent kinase inhibitor in patients with refractory solid tumors. ESMO meeting. Ann. Oncol..

[38] AT7519. http://clinicaltrials.gov/ct2/results?term=AT7519.

[39] Tong W.G., Chen R., Plunkett W., Siegel D., Sinha R., Harvey R.D., Badros A.Z., Popplewell L., Coutre S., Fox J.A. (2010). Phase I and pharmacologic study of SNS-032, a potent and selective CDK2, 7, and 9 inhibitor, in patients with advanced chronic lymphocytic leukemia and multiple myeloma. ASCO Annual Meeting. J. Clin. Oncol..

[40] Guha M. (2013). Blockbuster dreams for Pfizer’s CDK inhibitor. Nat. Biotechnol..

[41] Smolewski P. (2008). Terameprocol, a novel site-specific transcription inhibitor with anticancer activity. IDrugs.

[42] Phase 1 Study of Terameprocol (EM-1421) a Survivin and Cyclin-Dependent Kinase-1 (Cdc2) Inhibitor, in Patients with Leukemia. http://www.clinicaltrials.gov/ct2/results?term=EM-1421&Search=Search.

[43] Cirstea D., Hideshima T., Santo L., Eda H., Mishima Y., Nemani N., Hu Y., Mimura N., Cottini F., Gorgun G. (2013). Small-molecule multi-targeted kinase inhibitor RGB-286638 triggers P53-dependent and -independent anti-multiple myeloma activity through inhibition of transcriptional CDKs. Leukemia.

[44] Phase 1 Open-label, Dose-escalation Clinical Study of the Safety and Tolerability of RGB-286638, a Novel, Multi-targeted Kinase Inhibitor, Administered to Patients with Selected, Relapsed or Refractory Hematological Malignancies. http://clinicaltrials.gov/ct2/show/NCT01168882?term=RGB-286638&rank=1.

[45] Agennix.com. http://www.agennix.com.

[46] Kim K.S., Sack J.S., Tokarski J.S., Qian L., Chao S.T., Leith L., Kelly Y.F., Misra R.N., Hunt J.T., Kimball S.D. (2000). Thio- and Oxoflavopiridols, Cyclin-Dependent Kinase 1-Selective Inhibitors: Synthesis and Biological Effects. J. Med. Chem..

[47] Murthi K.K., Dubay M., McClure C., Brizuela L., Boisclair M.D., Worland P.J., Mansuri M.M., Pal K. (2000). Structure-activity relationship studies of flavopiridol analogues. Bioorg. Med. Chem. Lett..

[48] Joshi K.S., Rathos M.J., Mahajan P., Wagh V., Shenoy S., Bhatia D., Chile S., Sivakumar M., Maier A., Fiebig H.H. (2007). P276–00, a novel cyclin-dependent inhibitor induces G1-G2 arrest, shows antitumor activity on cisplatin-resistant cells and significant *in vivo* efficacy in tumor models. Mol. Cancer Ther..

[49] Siemeister G., Lucking U., Wengner A.M., Lienau P., Steinke W., Schatz C., Mumberg D., Ziegelbauer K. (2012). BAY 1000394, a novel cyclin-dependent kinase inhibitor, with potent antitumor activity in mono- and in combination treatment upon oral application. Mol. Cancer Ther..

[50] Lücking U., Jautelat R., Krüger M., Brumby T., Lienau P., Schäfer M., Briem H., Schulze J., Hillisch A., Reichel A. (2013). The Lab Oddity Prevails: Discovery of Pan-CDK Inhibitor (*R*)-S-Cyclopropyl-S-(4-{[4-{[(1*R*,2*R*)-2-hydroxy-1-methylpropyl]oxy}-5-(trifluoromethyl)pyrimidin-2-yl]amino}phenyl)sulfoximide (BAY 1000394) for the Treatment of Cancer. ChemMedChem.

[51] William A.D., Lee A.C.H., Blanchard S., Poulsen A., Teo E.L., Nagaraj H., Tan E., Chen D., Williams M., Sun E.T. (2011). Discovery of the Macrocycle 11-(2-Pyrrolidin-1-yl-ethoxy)-14,19-dioxa-5,7,26-triazatetracyclo[19.3.1.1(2,6).1(8,12)]heptacosa1(25),2(26),3,5,8,10,12(27),16,21, 23- decaene (SB1518), a Potent Janus Kinase 2/Fms-Like Tyrosine Kinase-3 (JAK2/FLT3) Inhibitor for the Treatment of Myelofibrosis and Lymphoma. J. Med. Chem..

[52] Goh K.C., Novotny-Diermayr V., Hart S., Ong L.C., Loh Y.K., Cheong A., Tan Y.C., Hu C., Jayaraman R., William A.D. (2012). TG02, a novel oral multi-kinase inhibitor of CDKs, JAK2 and FLT3 with potent anti-leukemic properties. Leukemia.

[53] Poulsen A., William A., Blanchard S., Nagaraj H., Williams M., Wang H., Lee A., Sun E., Teo E.-L., Tan E. (2013). Structure-based design of nitrogen-linked macrocyclic kinase inhibitors leading to the clinical candidate SB1317/TG02, a potent inhibitor of cyclin dependant kinases (CDKs), Janus kinase 2 (JAK2), and Fms-like tyrosine kinase-3 (FLT3). J. Mol. Model..

[54] Phase 1 Dose-Escalation and Pharmacokinetic Study of TG02 Citrate in Patients with Relapsed or Refractory Chronic Lymphocytic Leukemia and Small Lymphocytic Lymphoma. http://www.clinicaltrials.gov/ct2/show/NCT01699152?term=TG02&rank=2.

[55] Phase 1 Dose-Escalation and Pharmacokinetic Study of TG02 Citrate in Patients with Advanced Hematological Malignancies. http://www.clinicaltrials.gov/ct2/show/NCT01204164?term=TG02&rank=1.

[56] Phase II Study of Oral PHA-848125AC in Patients with Thymic Carcinoma Previously Treated with Chemotherapy. http://clinicaltrials.gov/show/NCT01011439.

[57] Besse B., Garassino M.C., Rajan A., Novello S., Mazieres J., Weiss G.J., Ciomei M., Martignoni M., Petroccione A., Davite C. A phase II study of milciclib (PHA-848125AC) in patients with thymic carcinoma. Proceedings of the American Society of Clinical Oncology Conference.

[58] Ciomei M., Scaburri A. (2010). CDK Inhibitor for the Treatment of Mesothelioma.

[59] Kurt S. (2014). LEE011 CDK Inhibitor Showing Early Promise in Drug-Resistant Cancers. Oncol. Times.

[60] Macmillan Publishers Limited (2014). CDK inhibitors speed ahead. Nat. Rev. Drug Discov..

[61] A Phase 1b Study of LY2835219 in Combination with Endocrine Therapies for Patients with Hormone Receptor Positive, HER2 Negative Metastatic Breast Cancer. http://clinicaltrials.gov/ct2/show/NCT02057133?term=LY2835219&rank=5.

[62] A Randomized, Double-Blind, Placebo-Controlled, Phase 3 Study of Fulvestrant with or without LY2835219, a CDK4/6 Inhibitor, for Women with Hormone Receptor Positive, HER2 Negative Locally Advanced or Metastatic Breast Cancer. http://clinicaltrials.gov/ct2/show/NCT02107703?term=LY2835219&rank=9.

[63] Kubo A., Nakagawa K., Varma R.K., Conrad N.K., Cheng J.Q., Lee W.C., Testa J.R., Johnson B.E., Kaye F.J., Kelley M.J. (1999). The p16 status of tumor cell lines identifies small molecule inhibitors specific for cyclin-dependent kinase 4. AACR meeting. Clin. Cancer Res..

[64] Liggett W.H., Sidransky D. (1998). Role of the p16 tumor suppressor gene in cancer. ASCO Annual Meeting. J. Clin. Oncol..

[65] Musgrove E.A., Caldon C.E., Barraclough J., Stone A., Sutherland R.L. (2011). Cyclin D as a therapeutic target in cancer. Nat. Rev. Cancer.

[66] Adams P.D., Sellers W.R., Sharma S.K., Wu A.D., Nalin C.M., Kaelin W.G. (1996). Identification of a cyclin-CDK2 recognition motif present in substrates and p21-like cyclin-dependent kinase inhibitors. Mol. Cell. Biol..

[67] Harper J.W., Adams P.D. (2001). Cyclin-Dependent Kinases. Chem. Rev..

[68] Gondeau C., Gerbal-Chaloin S., Bello P., Aldrian-Herrada G., Morris M.C., Divita G. (2005). Design of a novel class of peptide inhibitors of cyclin-dependent kinase/cyclin activation. J. Biol. Chem..

[69] Andrews M.J., McInnes C., Kontopidis G., Innes L., Cowan A., Plater A., Fischer P.M. (2004). Design, synthesis, biological activity and structural analysis of cyclic peptide inhibitors targeting the substrate recruitment site of cyclin-dependent kinase complexes. Org. Biomol. Chem..

[70] McInnes C., Andrews M.J., Zheleva D.I., Lane D.P., Fischer P.M. (2003). Peptidomimetic design of CDK inhibitors targeting the recruitment site of the cyclin subunit. Curr. Med. Chem. Anti-Cancer Agents.

[71] Liu S., Premnath P.N., Bolger J.K., Perkins T.L., Kirkland L.O., Kontopidis G., McInnes C. (2013). Optimization of Non-ATP Competitive CDK/Cyclin Groove Inhibitors through REPLACE-Mediated Fragment Assembly. J. Med. Chem..

[72] Pargellis C., Tong L., Churchill L., Cirillo P.F., Gilmore T., Graham A.G., Grob P.M., Hickey E.R., Moss N., Pav S. (2002). Inhibition of p38 MAP kinase by utilizing a novel allosteric binding site. Nat. Struct. Biol..

[73] Ohren J.F., Chen H., Pavlovsky A., Whitehead C., Zhang E., Kuffa P., Yan C., McConnell P., Spessard C., Banotai C. (2004). Structures of human MAP kinase kinase 1 (MEK1) and MEK2 describe novel noncompetitive kinase inhibition. Nat. Struct. Mol. Biol..

[74] Betzi S., Alam R., Martin M., Lubbers D.J., Han H., Jakkaraj S.R., Georg G.I., Schonbrunn E. (2011). Discovery of a potential allosteric ligand binding site in CDK2. ACS Chem. Biol..

[75] Ember S.W.J., Zhu J.-Y., Olesen S.H., Martin M.P., Becker A., Berndt N., Georg G.I., Schonbrunn E. (2014). Acetyl-lysine Binding Site of Bromodomain-Containing Protein 4 (BRD4) Interacts with Diverse Kinase Inhibitors. ACS Chem. Biol..

[76] Martin M.P., Olesen S.H., Georg G.I., Schonbrunn E. (2013). Cyclin-dependent kinase inhibitor dinaciclib interacts with the acetyl-lysine recognition site of bromodomains. ACS Chem. Biol..

